# Synthesis and Characterization of Some New Cu(II), Ni(II) and Zn(II) Complexes with Salicylidene Thiosemicarbazones: Antibacterial, Antifungal and *in Vitro* Antileukemia Activity

**DOI:** 10.3390/molecules18088812

**Published:** 2013-07-24

**Authors:** Elena Pahontu, Valeriu Fala, Aurelian Gulea, Donald Poirier, Victor Tapcov, Tudor Rosu

**Affiliations:** 1 Inorganic Chemistry Department, Faculty of Pharmacy, University of Medicine and Pharmacy “Carol Davila”, 6 Traian Vuia Street, 020956 Bucharest, Romania; 2 Dental Education Department, Moldova State University of Medicine and Pharmacy “N. Testemitsanu” Chisinau, Republic of Moldova; 3 Coordination Chemistry Department, Moldova State University, 60 Mateevici Street, 2009 Chisinau, Republic of Moldova; 4 Laboratory of Medicinal Chemistry, CHUQ (CHUL)- Research Center and Université Laval, 2705 Boulevard Laurier, Québec City, QC G1V 4G2 Canada; 5 Inorganic Chemistry Department, Faculty of Chemistry, University of Bucharest, 23 Dumbrava Rosie Street, 020462 Bucharest, Romania

**Keywords:** copper, nickel, zinc complexes, thiosemicarbazones, antimicrobial activity, antileukemia

## Abstract

Thirty two new Cu(II), Ni(II) and Zn(II) complexes (**1**–**32**) with salicylidene thiosemicarbazones (**H_2_L^1^**–**H_2_L^10^**) were synthesized. Salicylidene thiosemicarbazones, of general formula (X)N-NH-C(S)-NH(Y), were prepared through the condensation reaction of 2-hydroxybenzaldehyde and its derivatives (X) with thiosemicarbazide or 4-phenylthiosemicarbazide (Y = H, C_6_H_5_). The characterization of the new formed compounds was done by ^1^H-NMR, ^13^C-NMR, IR spectroscopy, elemental analysis, magnetochemical, thermoanalytical and molar conductance measurements. In addition, the structure of the complex **5** has been determined by X-ray diffraction method. All ligands and metal complexes were tested as inhibitors of human leukemia (HL-60) cells growth and antibacterial and antifungal activities.

## 1. Introduction

The design and study of well-arranged metal-containing Schiff bases with ONS – donor atoms is an interesting field of inorganic and bioinorganic chemistry [1,2,3,4,5,6,7,8,9,10,11]. *In-situ* one-pot template condensation reactions lie at the heart of the coordination chemistry. Transition metal complexes have also received great attention because of their biological interests, including antiviral, anticarcinogenic, antibacterial and antifungal activities [12,13,14,15,16]. Thiosemicarbazones and their Cu(II) complexes demonstrated potent cytotoxic activities against a series of murine and human tumor cells in culture [17,18,19].

In a recent study [20], we have concluded that the *in vitro* HL-60 leukemia cell growth inhibitory activity is influenced by the nature and geometric structure of copper complexes. Indeed, copper complexes containing tridentate ONS Schiff bases as well as salicyliden thiosemicarbazones have been found as effective inhibitors of cell proliferation. We have started a program directed toward the synthesis of different classes of anticancer, antibacterial and antifungal agents designed with complexes of a transition metal and an organic ligand [21,22,23,24].

In continuation of this approach, the present paper describes the synthesis, characterisation and *in vitro* evaluation of inhibitors of HL-60 cell proliferation, antibacterial and antifungal activity using thirty two novel Cu(II), Ni(II) and Zn(II) complexes with the salicylidene thiosemicarbazones (**H_2_L^1^**–**H_2_L^10^**), obtained from the condensation reaction of thiosemicarbazide or 4-phenylthiosemicarbazide with 2-hydroxybenzaldehyde derivatives. All ligands and metal complexes were tested as inhibitors of human leukemia (HL-60) cell growth. The Cu(II) complexes **21**–**25**, **30** have also been tested for their *in vitro* antibacterial activity against *Staphylococcus aureus (Wood-46, Smith, 209-P), Staphylococcus saprophyticus, Streptococcus (group A), Enterococcus faecalis* (Gram-positive)*, Escherichia coli (O-111), Salmonella typhimurium, Salmonella enteritidis, Klebsiella pneumoniaie, Pseudomonas aeruginosa, Proteus vulgaris and Proteus mirabilis* (Gram-negative) and antifungal activity against *Aspergillus niger, Aspergillus fumigatus, Candida albicans* and *Penicillium* strains.

## 2. Results and Discussion

### 2.1. Chemistry

The salicylidene thiosemicarbazones **H_2_L^1^**–**H_2_L^10^** used in this work were prepared by refluxing (for 30 min.) in ethanol an equimolar amount of aldehyde (salicylaldehyde or its derivatives, 5-chloro-, 5-bromo-, 5-nitro-, 5-methyl- and 3,5-dichlorosalicylaldehyde) and thiosemicarbazide or 4-phenylthiosemicarbazide. The structures of the Schiff bases **H_2_L^1^**–**H_2_L^10^** were established by IR, ^1^H-NMR and ^13^C-NMR spectroscopy.

These Schiff bases were further used for the complexation reaction with Cu^2+^, Ni^2+^, Zn^2+^ metal ions, using the following salts: CuSO_4_·5H_2_O (for complexes **1**–**7**), Cu(NO_3_)_2_·3H_2_O (for **8**–**14**), CuCl_2_·2H_2_O (for **15**–**30**), NiCl_2_·6H_2_O (for **31**) and ZnCl_2_ (for **32**). To metal salt (10 mmol) dissolved in distilled water was added salicylidene thiosemicarbazone, **HL**, (10 mmol) dissolved in ethanol. The reaction mixture was stirred and heated (50–55 °C) for 1.5 h. The precipitate was filtered, washed with ethanol, ether and dried in air.

The complexes obtained are microcrystalline solids which are stable in air and decompose above 310 °C (Table 1). They are insoluble in organic solvents such as acetone and chloroform but soluble in DMF and DMSO.

The molar conductance of the soluble complexes in DMF showed values indicating that **1**–**14** (80–100 ohm^−1^ cm^2^ mol^−1^) are electrolytes and **15**–**32** (10–20 ohm^−1^ cm^2^ mol^−1^) are non-electrolytes in nature [25].

**Scheme 1:**
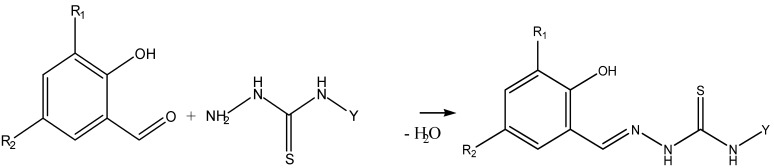
General synthesis of organic ligands **H_2_L^1−1^°**.

**Figure 1:**
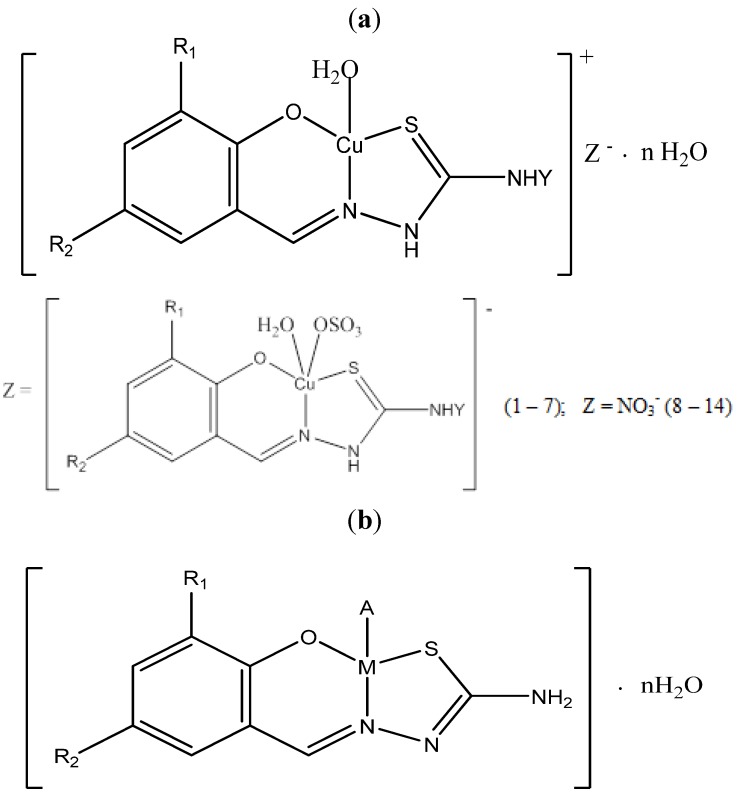
(**a**) General structure of complexes **1**–**14**. (**b**) General structure of complexes **15**–**32**.

The elemental analyses data of Schiff bases (reported in the Experimental section) and their complexes (Table 1) are in agreement with the proposed composition of the ligands as shown in Scheme 1 and with the formulas of the complexes as shown in Figure 1a,b.

R_1_ = H, R_2_ = H, Y = H **(H_2_L^1^)**, R_1_ = Cl, R_2_ = Cl, Y = H **(H_2_L^6^)**

R_1_ = H, R_2_ = Cl, Y = H **(H_2_L^2^)**, R_1_ = Br, R_2_ = Br, Y = H **(H_2_L^7^)**

R_1_ = H, R_2_ = Br, Y = H **(H_2_L^3^)**, R_1_ = H, R_2_ = H, Y = C_6_H_5_ **(H_2_L^8^)**

R_1_ = H, R_2_ = NO_2_, Y = H **(H_2_L^4^)**, R_1_ = H, R_2_ = Br, Y = C_6_H_5_ **(H_2_L^9^)**

R_1_ = H, R_2_ = CH_3_, Y = H **(H_2_L^5^)**, R_1_ = H, R_2_ = NO_2_, Y = C_6_H_5_ **(H_2_L^10^)**

M = Cu **(15**–**30)**, Ni **(31)**, Zn **(32);** R_1_ = H (**1**–**19**, **30**–**32**), Cl (**20**), Br (**21**–**29**); R_2_ = H (**1**, **2**, **8**, **9**, **15**, **30**–**32**), CH_3_ (**19**), Cl (7, **14**, **16**, **20**), Br (**5**, **6**, **12**, **13**, **17**, **21**–**29**), NO_2_ (**3**, **4**, **10**, **11**, **18**); Y = H (**1**, **3**, **5**, **7**, **8**, **10**, **12**, **14**–**32**), C_6_H_5_** (2**, **4**, **6**, **9**, **11**, **13**).

**Table:** 

A-Structure	Name	Complex
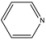	Py	15–20
**H_3_N**	-	21
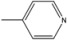	4-MePy	22
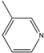	3-MePy	23
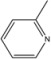	2-MePy	24
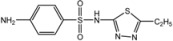	Etz	25, 30–32
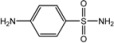	Str	26
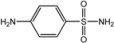	Sfc	27
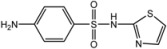	Nor	28
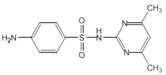	Sdm	29

**Table 1:** Physical and analytical data of the metal complexes **1**–**32 ^a^**.

Comp. No.	Molecular formula	Mr ^b^	µ _eff_^c^ B.M.	C, H, N, calc (found) %	M(3d) ^d^ %	IR (cm^−1^)	η, % ^e^	T, C ^f^ dec
**1**	C_16_H_24_Cu_2_N_6_O_10_S_3_ [Cu(H_2_O)(HL^1^)][Cu(H_2_O)(HL^1^)SO_4_] ^.^ 2H_2_O	684	2.14	C: 28.1(28.5); H: 3.5 (3.0); N: 12.3(12.5); S: 14.0 (13.7)	18.7 (18.6)	H_2_O (3585, 1575, 920); NH_2_ (3435, 3420); NH(3335, 3220, 3145); C= N (1605); C-O (1200); C = S (781); Cu-N (510, 415); Cu-O (470); Cu-S (450)	65	460
**2**	C_28_H_30_Cu_2_N_6_O_9_S_3_ [Cu(H_2_O)(HL^8^)][Cu(H_2_O)][(HL^8^)SO_4_] ^.^ H_2_O	818	2.07	C: 41.1(41.4); H: 3.7 (3.4); N: 10.3(10.3); S: 11.7 (11.6)	15.6 (15.8)	H_2_O (3580, 1570, 925); NH (3325, 3222, 3143); C = N (1600); C-0(1195); C = S (780); Cu-N(517, 428); Cu-O (472); Cu-S (445)	64	450
**3**	C_16_H_22_Cu_2_N_8_O_14_S_3_ [Cu(H_2_O)(HL^4^)][Cu(H_2_O)(HL^4^)(SO_4_) ] ^.^ H_2_O	774	1.98	C: 24.8 (24.5); H: 2.8(2.7); N: 14.5 (14.8); S: 12.4 (12.7)	16.5 (16.3)	H_2_O (3575, 1570, 922); NH_2_ (3445, 3425); NH (3330 3230, 3140); C = N (1590); C-O (1195); C = S (776); Cu-N (530, 410); Cu-O (470); Cu-S (465)	77	425
**4**	C_28_H_30_Cu_2_N_8_O_14_S_3_ [Cu(H_2_O)(HL^1^°)][Cu(H_2_O)(HL^1^°)(SO_4_)]^.^ 2H_2_O	926	2.09	C: 36.3 (36.5); H: 3.2 (3.0); N: 12.1 (12.4); S: 10.4 (10.1)	13.8 (14.1)	H_2_O (3580, 1583, 915); NH (3315, 3230, 3138); C = N (1585); C-O (1197); C = S (779); Cu-N (525, 430); Cu-O (475); Cu-S(440)	72	410
**5**	C_16_H_26_Br_2_Cu_2_N_6_O_12_S_3_ [Cu(H_2_O)(HL^3^)][Cu(H_2_O)(HL^3^)(SO_4_)] ^.^ 4H_2_O	878	1.85	C: 21.9 (22.2); H: 3.3 (3.3); Br: 18.2 (18.4); N: 9.6 (9.4); S: 10.3 (10.5)	14.6 (14.4)	H_2_O (3565, 1575, 935); NH_2_ (3445, 3430); NH (3340, 3230, 3137); C = N (1590); C-O (1205); C = S (780); Cu-N (505, 430); Cu-O (485); Cu-S (462)	69	450
**6**	C_28_H_28_Br_2_Cu_2_N_6_O_9_S_3_ [Cu(H_2_O)(HL^9^)][Cu(H_2_O)(HL^9^)(SO_4_)] ^.^ H_2_O	976	1.91	C: 34.4 (34.0); H: 2.9 (2.7); Br: 16.4 (16.5); N: 8.6 (8.4); S: 9.8 (9.9)	13.1 (12.8)	H_2_O (3580, 1565, 930); NH (3330, 3225, 3145); C = N (1585); C-O (1203); C = S (778); Cu-N (525, 425); Cu-O (484); Cu-S (465)	56	435
**7**	C_16_H_20_Cl_2_Cu_2_N_6_O_9_S_3_ [Cu(H_2_O)(HL^2^)][Cu(H_2_O)(HL^2^)(SO_4_)] ^.^ H_2_O	735	1.79	C: 26.1 (26.3); H: 2.7 (2.4); Cl: 9.7 (10.0); N: 11.4 (11.5); S: 13.1 (13.3)	17.4 (17.7)	H_2_O (3585, 1575, 920); NH_2_ (3430, 3430); NH (3335, 3220, 3145); C = N (1595); C-0(1200); C = S(785); Cu-N (528, 410); Cu-O (482); Cu-S (464)	78	430
**8**	C_8_H_12_CuN_4_O_6_S [Cu(H_2_O) (HL^1^)]NO_3_^.^ H_2_O	356	1.87	C: 27.0 (27.3); H: 3.4 (3.1); N: 15.7 (15.5); S: 9.0 (9.4)	18.0 (18.2)	H_2_O (3580, 1574, 915); NH_2_ (3440, 3430); NH (3325, 3230, 3140); C = N(1600); C-0(1200); C = S(776); Cu-N(530, 410); Cu-O(480); Cu-S(450)	70	390
**9**	C_14_H_16_CuN_4_O_6_S [Cu (H_2_O)(HL^8^)]NO_3_^.^ H_2_O	432	2.12	C: 38.9 (38.4); H: 3.7 (3.5); N: 13.0 (13.1); S: 7.4 (7.2)	14.8 (14.5)	H_2_O (3576, 1570, 930); NH(3345, 3227, 3146); C = N(1595); C-O (1198); C = S (787); Cu-N (525, 430); Cu-O (465); Cu-S (440)	54	380
**10**	C_8_H_11_CuN_5_O_8_S [Cu (H_2_O)(HL^4^)]NO_3_^.^ H_2_O	401	1.85	C: 23.9 (24.2); H: 2.7 (2.5); N: 17.5 (17.1); S: 8.0 (8.3)	16.0 (16.3)	H_2_O (3570, 1565, 925); NH_2_ (3445, 3430); NH (3325, 3215, 3140); C = N (1598); C-O (1195); C = S (777); Cu-N (525, 410); Cu-O (475); Cu-S (440)	76	325
**11**	C_14_H_17_CuN_5_O_9_S [Cu (H_2_O)(HL^1^°)]NO_3_^.^ 2H_2_O	495	1.94	C: 33.9 (34.1); H: 3.4 (3.5); N: 14.1 (14.2); S: 6.5 (6.9)	12.9 (12.7)	H_2_O (3590, 1585, 915); NH(3325, 3225, 3140); C = N(1593); C-0(1192); C = S(783); Cu-N(525, 430); Cu-O(480); Cu-S(455)	80	315
**12**	C_8_H_11_BrCuN_4_O_6_S [Cu (H_2_O)(HL^3^)]NO_3_^.^ H_2_O	435	1.80	C: 22.1 (21.8); H: 2.5 (2.2); N: 12.9 (13.2); S: 7.4 (7.5)	14.7 (14.5)	H_2_O (3585, 1575, 920); NH_2_(3430, 3415); NH(3335, 3220, 3145); C = N(1595); C-0(1195); C = S(784); Cu-N(525, 425); Cu-O(475); Cu-S(460)	52	370
**13**	C_14_H_19_BrCuN_4_O_8_S [Cu(H_2_O)(HL^9^)]NO_3_^.^ 3H_2_O	547	1.97	C: 30.7 (30.9); H: 3.4 (3.2); Br: 14.6 (14.4); N: 10.2 (9.9); S: 5.8 (5.7)	11.7 (11.5)	H_2_O (3570, 1565, 925); NH(3330, 3210, 3135); C = N(1590); C-0(1197); C = S(780); Cu-N(530, 423); Cu-O(470); Cu-S(465)	65	360
**14**	C_8_H_11_ClCuN_4_O_6_S [Cu(H_2_O)(HL^2^)]NO_3_^.^ H_2_O	390.5	2.03	C: 24.6 (24.6); H: 2.8 (2.5); Cl: 9.1 (9.3); N: 14.3 (14.5); S: 8.2 (8.5)	16.4 (16.1)	H_2_O (3585, 1575, 920); NH_2_(3435, 3425); NH(3335, 3220, 3145); C = N(1605); C-0(1193); C = S(780); Cu-N(515, 430); Cu-O(490); Cu-S(455)	75	365
**15**	C_13_H_12_CuN_4_OS [Cu L^1^Py]	336	1.78	C: 46.4 (46.5); H: 3.6 (3.5); N: 16.7 (16.5); S: 9.5 (9.4)	19.0 (18.8)	NH_2_(3440, 3425); C = N(1590, 1580, 1575, 1310); C-0 (1215); C-S ( 760); Cu-N(530, 425); Cu-O(475); Cu-S(460)	71	460
**16**	C_13_H_11_ClCuN_4_OS [Cu L^2^Py]	370.5	1.78	C: 42.1 (42.0); H: 3.0 (2.9); Cl: 9.6 (9.5); N: 15.1 (15.0); S: 8.6 (8.4)	17.3 (17.0)	NH_2_ (3435, 3420); C = N (1585, 1580, 1570, 1305); C-O (1225); C-S (750); Cu-N (510, 405); Cu-O (470); Cu-S (465)	72	440
**17**	C_13_H_11_BrCuN_4_OS [Cu L^3^Py]	415	1.93	C: 37.6 (37.5); H: 2.7 (2.5); Br: 19.3 (19.0); N: 13.5 (13.3); S: 7.7 (7.5)	15.4 (15.5)	NH_2_ (3430, 3420); C = N (1585, 1580, 1575, 1300); C-O (1210); C-S (750); Cu-N (515, 410); Cu-O (475); Cu-S (465)	75	450
**18 **	C_13_H_11_CuN_5_O_3_S [Cu L^4^Py]	381	1.84	C: 40.9 (40.8); H: 2.9 (2.8); N: 18.4 (18.2); S: 8.4 (8.3)	16.8 (16.7)	NH_2_ (3440, 3425); C = N (1585, 1580, 1570, 1315); C-O (1220); C-S ( 770); Cu-N (525, 410); Cu-O (470); Cu-S (465)	69	400
**19 **	C_14_H_14_CuN_4_OS [Cu L^5^Py]	350	1.75	C: 48.0 (47.8); H: 4.0 (3.9); N: 16.0 (15.9); S: 9.1 (9.0)	18.3 (18.0)	NH_2_ (3430, 3425); C = N (1590, 1585, 1570, 1315); C-O (1220); C-S ( 770); Cu-N (520, 415); Cu-O (470); Cu-S (465)	70	460
**20 **	C_13_H_10_Cl_2_CuN_4_OS [Cu L^6^Py]	405	1.80	C: 38.5 (38.3); H: 2.5 (2.4); Cl: 17.5 (17.4); N: 13.8 (13.6); S: 7.9 (7.8)	15.8 (15.7)	NH_2_ (3435, 3425); C = N (1585, 1580, 1575, 1305); C-O (1205); C-S ( 770); Cu-N (515, 430); Cu-O (485); Cu-S (47 0)	76	410
**21 **	C_8_H_12_Br_2_CuN_4_O_3_S [Cu L^7^(NH_3_)] × 2H_2_O	468	1.87	C: 20.5 (20.4); H: 2.6 (2.5); Br: 34.2 (34.0); S: 6.8(6.7)	11.9(1.7)	NH_2_ (3440, 3425); NH (3330, 3215, 3150); C = N (1582, 1585); C-O (1225); C-S (748); Cu-N (540, 425); Cu-O (490); Cu-S (410);	78	310
**22**	C_14_H_14_ Br_2_CuN_4_O_2_S [Cu L^7^(4-MePy)] × H_2_O	526	1.79	C: 31.9 (31.8); H: 2.7 (2.5); Br: 31.5(31.3); S: 6.3(6.0)	12.2(12.3)	NH_2_ (3435,3430); C = N (1580,1585); C-O (1225); CNC (1042); C-S (748); Cu-O (540); Cu-O (490); Cu-S (410)	77	380
** 23**	C_14_H_12_ Br_2_CuN_4_OS [Cu L^7^(3-MePy)]	508	1.99	C: 33.1 (33.0); H: 2.4 (2.2); Br: 31.5(31.4); S: 6.3(6.1)	12.6(12.4)	NH_2_ (3440,3425); C = N (1580,1585); C-O (1225); CNC (1042); C-S (748); Cu-O (540); Cu-O (490); Cu-S (410)	76	390
**24**	C_14_H_12_ Br_2_CuN_4_OS [Cu L^7^(2-MePy)]	508	1.92	C: 33.1 (32.9); H: 2.4 (2.5); Br: 31.5(31.4); S: 6.3(6.0)	12.6(12.3)	NH_2_ (3440,3430); C = N (1580,1585); C-O (1225); CNC (1042); C-S (748); Cu-O (540); Cu-O (490); Cu-S (410)	71	345
**25**	C_18_H_17_Br_2_CuN_7_O_3_S_3_ [Cu L^7^(Etz)]	699	1.35	C: 30.9 (31.0); H: 2.4 (2.2); Br: 22.9 (22.7); N: 14.0(13.9); S: 13.7(13.5)	9.2(8.6)	NH_2_ (3435,3425, 3420, 3410); C = N (1610, 1600, 1585); SO_2_ (1320, 1140), C-O (1215); Cu-N (540, 415); Cu-O (490); Cu-S (440)	81	470
**26**	C_14_H_13_Br_2_CuN_5_O_3_S_2_ [Cu L^7^(Str)]	587	1.28	C: 28.6 (28.5); H: 2.2 (2.0); Br: 27.3 (27.0); N: 11.9 (12.0); S: 10.9 (10.7)	10.9 (11.0)	NH_2_ (3415,3420,3405,3415); C = N (1600, 1585); SO_2_ (1325, 1140); C-O (1210); Cu-N (525, 410); Cu-O (475); Cu-S (455)	68	430
**27**	C_16_H_15_Br_2_CuN_5_O_4_S_2_ [Cu L^7^(Sfc)]	629	1.31	C: 30.5 (30.3); H 2.4 (2.5); Br: 25.4 (25.3); N: 11.1 (11.0); S: 10.2 (10.0)	10.2 (10.1)	NH_2_ (3420,3415,3415,3405); C = N (1605, 1590); SO_2_ (1320, 1145); C-O (1215); Cu-N (530, 425); Cu-O (480); Cu-S (465);	63	450
**28**	C_17_H_14_Br_2_CuN_6_O_3_S_3_ [Cu L^7^(Nor)]	670	1.35	C: 30.4 (30.2); H: 2.1 (2.0); Br: 23.9 (24.0); N: 12.5 (12.3); S: 14.3 (14.2)	9.6 (9.5)	NH_2_ (3430, 3425, 3415, 3410); C = N (1610, 1605, 1590); SO_2_ (1315, 1145); C-O (1210); Cu-N (530, 420); Cu-O (480); Cu-S (455);	69	470
**29**	C_20_H_19_Br_2_CuN_7_O_3_S_2_ [Cu L^7^(Sdm)]	693	1.22	C: 34.6 (34.5); H: 2.7 (2.5); Br: 23.1 (23.0); N: 14.1 (14.0); S: 9.2 (9.0)	9.2 (9.1)	NH_2_ (3440, 3430, 3425, 3415); C = N (1610, 1600, 1595); SO_2_ (1310, 1150); C-O (1215); Cu-N (510, 425); Cu-O (475); Cu-S (450);	68	460
**30**	C_18_H_19_CuN_7_O_3_S_3_ [Cu L^1^(Etz)]	541	1.45	C: 39.9 (40.0); H: 3.5 (3.4); N: 18.1 (17.9); S: 17.7(17.5)	11.8(11.6)	NH_2_ (3435, 3430, 3425, 3415); C = N (1600, 1595, 1590); SO_2_ (1310, 1140); C-O (1225); Cu-N (515, 410); Cu-O (470); Cu-S (465)	70	500
**31**	C_18_H_23_NiN_7_O_5_S_3_ [Ni L^1^(Etz)] × 2H_2_O	572	dia	C: 37.8 (37.5); H: 4.0 (3.8); N: 17,1 (17.0); S: 16.8 (16.6)	10.3(10.2)	NH_2_ (3430, 3430, 3420, 3410); C = N (1605, 1595, 1590); SO_2_ (1315, 1145); C-O (1220); Cu-N (525, 415); Cu-O (475); Cu-S (460)	80	380
**32 **	C_18_H_19_N_7_O_3_S_3_Zn [Zn L^1^(Etz)]	542	dia	C: 39.9 (40.0); H: 3.5 (3.4); N: 18.1 (18.0); S: 17.7 (17.5)	12.0 (11.8)	NH_2_ (3430, 3430, 3420, 3415); C = N (1605, 1595, 1585); SO_2_ (1315, 1140); C-0 (1215); Cu-N (525, 425); Cu-O (480); Cu-S (470)	75	490

^a^**H_2_L^10^**, used in the preparation of complexes are reported in Scheme 1. ^b^ Mr: relative molecular mass. ^c^ µ eff: magnetic moment. ^d^ M (3d): metal 3d. ^e^ η: yield. ^f^ T_dec_ .: decomposition temperature.

#### 2.1.1. X-ray Structure of [Cu(H_2_O)(HL^3^)][Cu(H_2_O)(HL^3^)(SO_4_)]·4H_2_O **(5)**

The structure of crystals, obtained from ethanolic solution after recrystallization of (**5**), has been determined by means of X-ray analysis and is similar to the structure described in [26].

#### 2.1.2. IR Spectra and Coordination Mode

The tentative assignments of the significant IR spectral bands of **H_2_L^1^**–**H_2_L^10^** and their Cu(II), Ni(II) and Zn(II) complexes are presented in Table 1. It has been established that the substituted salicylaldehyde thiosemicarbazones of complexes **1**–**14** behave as monodeprotonated tridentate ligands and are coordinated to the central ions through deprotonated phenolic oxygen atom, azomethinic nitrogen atom and sulphur atom forming five- and six-membered metalocycles [9,20,21].

The IR spectra of the free ligands shows a broad band at *ca*. 3600 cm^−1^ attributed to phenolic group, δ(OH). This band disappeared from IR spectra of complexes **1**–**14** [22,23,27]. Moreover, this is confirmed by the shift of ν(C-O) stretching vibration bands observed in the range of 1250-1240 cm^−1^ in the spectra of the free ligands, to lower frequency at around 1225–1210 cm^−1^ in the spectra of the complexes. This is further confirmed by the presence of the band appearing in the region 500-470 cm^−1^ assigned to the ν(M-O) frequency [28].

Likewise, the IR spectra of the ligands exhibits a strong band in the range 1620–1610 cm^−1^ assignable to ν(C = N). In the spectra of the complexes **1**–**14** this band is shifted to lower frequencies by ca. 25–15 cm^−1^ suggesting the coordination of the azomethine nitrogen to the central metal atom. Also, this coordination is supported of ν(M-N) vibration around 515–540 cm^−1^ [29].

In the IR spectra of the **H_2_L^1^**–**H_2_L^10^**, the ν(S-H) band at 2570 cm^−1^ [30,31,32,33] was absent, but the ν(C = S) bands at about 1560 and 822 cm^−1^ were present. These bands were shifted to lower wavenumbers in complexes **1**–**14** and this shift can be assigned to the thiocarbonyl ν(C = S) stretching and bending modes of vibrations and to the coordination of sulfur atom to metal ion [34,35,36].

In complexes **15**–**32**, thiosemicarbazones behave as double deprotonated tridentate ligands, coordinating to the central ion through phenolic oxygen atom, azomethinic nitrogen atom and sulphur atom forming two five- and six-membered heterocycles. As much, the absorption bands ν(C-OH), ν(N-NH) and ν(C=S), observed in the spectra of the free thiosemicarbazones, in the range 1245–1240, 1540–1535 and 1125–1120 cm^−1^, respectively, were shifted to lower frequencies in the spectra complexes. In the spectra complexes the absorption band ν(C-S) is observed in the range 750–740 cm^−1^ and the band ν(C-N) is shifted to small frequencies with 35-30 cm^−1^, being accompanied by the splitting into two components [27,28,29].

In the IR spectra of complexes **15**–**32**, an absorption band is observed in the range 1520–1518 cm^−1^, conditioned by valence oscillations >C = N-N = C<. This character of IR spectra demonstrates the thiosemicarbazone enolization in the process of synthesized complexes formation [30,31,32,33]. 

The nitrate complexes **8**–**14** shows a single band at around 1345-1340 cm^−1^. It is attributable to ionic NO_3_^−^ [37].

In compounds **1**–**14** the absorption bands characteristic to the water molecule from the inner sphere are observed: ν(H_2_O) = 3595–3585 cm^−1^, δ(H_2_O) = 1590–1585 cm^−1^, γ(H_2_O) = 920–915 cm^−1^, *w*(H_2_O) = 640–615 cm^−1^ due to OH stretching, HOH deformation, H_2_O rocking and H_2_O wagging, respectively [38].

The presence of sulphanilamides in complexes **25**–**32** is confirmed by the characteristic absorption bands observed in IR spectra: ν_as_(NH_2_), ν_s_(NH_2_): ≈ 3400 cm−1; ν(N-H): 3330 ± 20 cm−1, ν(C-N)_(arom)_: 1305 ± 55 cm−1, ν(C = N)_(arom)_ 1580 ± 30 cm−1; ν_as_(SO_2_), ν_s_(SO_2_): 1320 ± 20 cm−1, 1100 ± 20 cm−1. It has been established that the investigated sulphanilamides of the given complexes behave as monodentate ligands and are coordinated to the central atom through nitrogen atoms and amino groups in the case of streptocide (Str) and sulphacil (Sfc), thiadiazolic nitrogen atom in the case of ethazole (Etz) and norsulphazole (Nor) one of the pyrimidinic nitrogen atoms in the case of sulphadimezine (Sdm) [38].

#### 2.1.3. Magnetochemistry

The room temperature magnetic moment of the solid copper (II) complexes **1-24** was found in the range 1.75–2.00 BM, indicative one unpaired electron per Cu(II) ion [39]. These experimental data allow us to suppose that in these compounds the spin-spin interaction lacks and probably the investigated complexes have monomer structure. Also, the magnetic moment values in the range 1.22–1.45 BM for the copper (II) complexes **25**–**30** are of indicative anti-ferromagnetic spin-spin interaction through molecular association [40]. Complex **31** is diamagnetic and the central Ni^2+^ ion is in a square planar environment [40].

#### 2.1.4. Thermal Decomposition

All complexes studied were investigated by thermogravimetry analysis. The TG thermograms of complexes **1**–**14** are characterized by three degradation steps (50–100, 130–170, 310–530 °C). The weight loss between 50 and 100 °C corresponds to the elimination of water molecules of dehydration and is an endothermic effect. The second step, also an endothermic effect, corresponds to the elimination of coordinated water molecules (Table 1). The following effect on DTA curve is exothermic and corresponds to the complete decomposition (TG, TGD curves) of the organic part of the complexes.

The TG and TGD curves of the complexes **15**–**32** are characterized by two steps of weight loss united (350–480 °C, 480–620 °C) and corresponds to the complete decomposition of the ligands. In addition, the TG and TGD curves of the complexes **21**, **22** and **31** are characterized by a weight loss in the renge 50–100 °C.

By replacing the sulphate ion from complexes with nitrate ion or by changing the thiosemicarbazide fragment with 4-phenylthiosemicarbazide fragment, TG and TGD curves show weight loss at lower temperatures. The final residues were identified by IR spectroscopy as CuO, which provides %Cu values in the initial samples, by quantitative analyses. They were in agreement wich the theoretical obtained %Cu values. 

#### 2.1.5. NMR Spectra

The NMR spectra of ligands **H_2_L^1^**–**H_2_L^10^** were recorded in DMSO-d_6_. The ^1^H-NMR and ^13^C-NMR spectral data are reported along with the possible assignments [41]. All the protons were found to be in the expected regions. It was observed that DMSO did not have any coordinating effect on the ligands or their metal complexes.

#### 2.1.6. Mass Spectra

The FAB mass spectra of Cu(II), Ni(II) and Zn(II) complexes with salicyliden thiosemicarbazones (**H_2_L^1^**–**H_2_L^10^**) have been recorded (Table 2). The molecular ion [M]^+^ peaks obtained from Cu(II), Ni(II) and Zn(II) complexes are as follows: *m/z* = 274.8 (**1**), *m/z* = 319.7 (**3**), *m/z* = 309.6 (**7**), *m/z* = 350.9 (**9**), *m/z* = 395.6 (**11**), *m/z* = 429.8 (**13**), *m/z* = 369.8 (**16**), *m/z* = 349.3 (**19**), *m/z* = 506.8 (**22**), *m/z* = 698.2 (**25**), *m/z* = 586.1 (**26**), *m/z* = 536 (**31**), *m/z* = 541.4 (**32**). The data obtained are in good agreement with the proposed molecular formula for Cu(II), Ni(II) and Zn(II) complexes. The FAB mass spectra of these complexes shows peaks assignable to various fragments arising from the thermal cleavage of the complexes. 

**Table 2:** FAB mass spectral data of Cu(II) Ni(II) and Zn(II) complexes.

Molecular formula	Mw (g/mol)	Molecular ion peak [M]^+^	The peaks due to complex fragmentation			
[Cu(H_2_O)(HL^1^)][Cu(H_2_O)(HL^1^)SO_4_] . 2H_2_O (**1**)	684	274.8	101.2	170.3	203.4	
[Cu(H_2_O)(HL^4^)][Cu(H_2_O)(HL^4^)(SO_4_)] . H_2_O (**3**)	774	319.7	147.3	216.5	296.3	
[Cu(H_2_O)(HL^2^)][Cu(H_2_O)(HL^2^)(SO_4_)] ^.^ H_2_O (**7**)	735	309.6	136.7	206.3	287.5	
[Cu (H_2_O)(HL^8^)]NO_3_ . H_2_O (**9**)	432	350.9	101.7	171.4	203.8	320.2
[Cu (H_2_O)(HL^1^°)]NO_3_ . 2H_2_O (**11**)	495	395.6	147.7	220.2	286.3	372.1
[Cu(H_2_O)(HL^9^)]NO_3_ . 3H_2_O (**13**)	547	429.8	181.2	229.1	295.2	398.8
[Cu L^2^Py] (**16**)	370.5	369.8	136.7	207.5	292.1	322.6
[Cu L^5^Py] (**19**)	350	349.3	132.1	203.3	289.2	318.5
[Cu L^7^(4-MePy)] × H_2_O (**22**)	526	506.8	262.3	327.8	403.2	498.8
[Cu L^7^(Etz)] (**25**)	699	698.2	296.3	357.5	434.4	544.2
[Cu L^7^(Str)] (**26**)	587	586.1	284.1	345.6	422.1	532.4
[Ni L^1^(Etz)] × 2H_2_O (**31**)	572	536	269.5	330.6	401.3	517.8
[Zn L^1^(Etz)] (**32**)	542	541.4	282.2	344.2	416.1	527.4

### 2.2. Biological Activity

#### 2.2.1. Antiproliferative Activity of Human Leukemia HL-60 Cells

All ligands (Table 3) and their metal complexes (Table 4) were tested as inhibitors of HL-60 cells proliferation using three concentrations: 0.1, 1.0 and 10 μmol/L. At 0.1 and 1.0 μmol/L the ligands have unsignificant inhibitor activity, but at 10 μmol/L **H_2_L^8^** (salicylidene-4-phenylthiosemicarbazone), **H_2_L^9^** (5-Br-salicylidene-4-phenylthiosemicarbazone) and **H_2_L^1^** (5-NO_2_-salicyliden-4-phenylthio-semicarbazone) inhibit the cell proliferation (90, 75 and 70%, respectively). So, we can assert that the presence of phenyl-radical in the Schiff bases composition is important. The same fact is confirmed for copper complexes, but in the enforced variant. So, copper complexes act selectively in this biological process [23,42,43,44]. In fact, copper complexes, including inner sphere water and tridentate ONS ligands, are more active than those containing inner sphere amine, which blocked the metal active centre. Complexes **1–14** are thus better inhibitors of cell proliferation than complexes **15**–**30**.

If copper is capsulated with amine, the antiproliferative activity change in dependence of substituents R_1_ and R_2_ in the same series Y = H or Y = -C_6_H_5_. The following three examples illustrate our SAR results. If A = Py, Y = H and R_1_ = H, the antiproliferative activity varies (from 60% to 10%) depending on R_2_: H (**15**) > CH_3_ (**19**) > Br (**17**) > Cl (**16**) > NO_2_ (**18**). If Y = H, R_1_ = R_2_ = Br and A - is variable, the moderate influence of amine nature can be observed depending on the ability of amine(N)-copper bond force: **25** > **28** > **26** **=** **27** = **29** **>** **23** = **24** **>** **21** **>** **22**. If Y = H, R_1_ = R_2_ = H, A = ethazole and copper ion is replaced by nickel or zinc (**31**, **32**), the antiproliferative activity dramatically decreases.

**Table 3:** Schiff bases **H_2_L^1^**–**H_2_L^10^** and their antiproliferative activity on human leukemia (HL-60) cells at three concentrations.

Schiff base	(X)N-NH-C(S)-NH(Y)			Inhibition of cell proliferation (%)		
	X		Y	10 µM	1 µM	0.1µM
	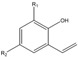					
	R_1_	R_2_				
H_2_L^1^	H	H	H	20	10	0
H_2_L^2^	H	Cl	H	0	0	0
H_2_L^3^	H	Br	H	5	0	0
H_2_L^4^	H	NO_2_	H	0	0	0
H_2_L^5^	H	CH_3_	H	5	0	0
H_2_L^6^	Cl	Cl	H	10	0	0
H_2_L^7^	Br	Br	H	0	0	0
H_2_L^8^	H	H	C_6_H_5_	90	0	0
H_2_L^9^	H	Br	C_6_H_5_	75	0	0
H_2_L^10^	H	NO_2_	C_6_H_5_	70	0	0

SEM < ± 4% of a single experiment in triplicate.

**Table 4:** Antiproliferative activity of complexes **1**–**32** on human leukemia (HL-60) cells at three concentrations.

Complex ^a^	Structural formula of copper complex			Inhibition of cell proliferation (%) ^b^			Complex ^a^	Structural formula of metal complexes			Inhibition of cell proliferation (%)^b^		
	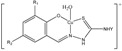			10 µM	1 µM	0.1 µM		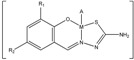			10 µM	1 µM	0.1 µM
	R_1_	R_2_	Y					R_1_	R_2_	A			
1	H	H	H	98	50	0	15	H	H	Py	-	35	10
2	H	H	-C_6_H_5_	100	90	0	16	H	Cl	Py	-	25	5
3	H	NO_2_	H	90	70	0	17	H	Br	Py	-	50	0
4	H	NO_2_	-C_6_H_5_	96	78	0	18	H	NO_2_	Py	-	10	0
5	H	Br	H	95	90	0	19	H	CH_3_	Py	-	55	0
6	H	Br	-C_6_H_5_	90	90	0	20	Cl	Cl	Py	-	60	10
7	H	Cl	H	95	95	0	21	Br	Br	NH_3_	-	25	0
8	H	H	H	100	95	0	22	Br	Br	4-MePy	-	20	0
9	H	H	-C_6_H_5_	100	100	0	23	Br	Br	3-MePy	-	30	15
10	H	NO_2_	H	100	90	0	24	Br	Br	2-MePy	-	30	5
11	H	NO_2_	-C_6_H_5_	100	90	0	25	Br	Br	Ethazole	-	60	15
12	H	Br	H	98	95	0	26	Br	Br	Streptocide	65	40	5
13	H	Br	C_6_H_5_	100	80	0	27	Br	Br	Sulfocile	65	40	5
14	H	Cl	H	100	90	0	28	Br	Br	Norsulfosole	65	55	5
							29	Br	Br	Sulfadimizine	65	40	5
DOX				100	100	30	30	H	H	Ethazole	60	65	0
							31	H	H	Ethazole	5	5	5
							32	H	H	Ethazole	10	5	0

^a^ The molecular formula of complexes are reported in Table 1. ^b^ SEM < ± 4% of a single experiment in triplicate. DOX = Doxorubicine.

#### 2.2.2. Antibacterial Activity

Experimental results obtained from the study of antimicrobial activity (Table 5) demonstrate that compounds **21**–**25** and **30** display bacteriostatic and bactericide activity in the concentration range 0.03-4000 µg/mL towards both Gram-positive as well as Gram-negative bacteria. In comparison, the antimicrobial data characteristic for *furacillinum* used in medical practice are given. The antimicrobial activity displayed by the above mentioned compounds is 32–260 times higher towards staphylococcus and streptococcus than *furacillinum* and exceeds by 2–260 times her bacteriostatic activity towards the majority of Gram-negative bacteria. The minimum inhibitory concentration (MIC) and minimum bactericide concentration (MBC) are influenced by the nature of thiosemicarbazone and amine of the inner sphere of the coordination compound.

The data concerning the study of antimycotic properties of compounds **22**–**24** show that they also display selective bacteriostatic and bactericide activity in the concentration range 9.3–600 μg/mL towards investigated fungi stems. In order to make a comparison, we also added data regarding the activity of nystatine, a compound used in medicine at mycoses treatment. The results show that the synthesized substances have antimycotic activity against most fungi, higher than nystatine activity. Aspergillus fumigatus is an exception, being less sensible towards mentioned substances. The toxicity (LD_50_) of complexes **24** and **30** (some of the most active in this group of substances) is 1,420 mg/kg and 4,250 mg/kg so it is 8.6–25.5 times lower than that of furacillinum (LD_50_ = 166.7 mg/kg).

**Table 5:** Antimicrobial or antifungal activity (MIC ^a^/MBC ^b^) (μg/mL) of some copper complexes.

Stem			Complexes ^c^							
			21	22	23	24	25	30	Furacillinum	Nystatin
*Staphylococcus* *aureus*	Wood-46	MIC	0.29	0.145	0.145	0.29	0.06	0.03	9.35	-
		MBC	0.29	0.145	0.145	0.29	0.06	0.03	9.35	-
	Smith	MIC	0.29	0.145	0.29	0.29	-	-	9.35	-
		MBC	0.58	0.29	0.29	0.58	-	-	9.35	-
	209-P	MIC	0.58	0.29	0.29	0.29	0.06	0.03	18.7	-
		MBC	0.58	1.16	1.16	0.58	0.06	0.03	18.7	-
*Staphylococcus saprophyticus*		MIC	0.29	0.29	0.145	0.29	0.12	0.03	9.35	-
		MBC	0.29	0.58	0.145	0.29	0.24	0.06	18.7	-
*Streptococcus*( group A)		MIC	0.036	0.009	1.16	0.29	0.12	0.06	-	-
		MBC	0.072	0.036	2.33	0.58	0.24	0.06	-	-
*Enterococcus faecalis*		MIC	-	-	-	-	0.06	0.03	37.5	-
		MBC	-	-	-	-	0.06	0.097	37.5	-
*Escherichia coli*, O-111		MIC	1.16	9.35	18.7	4.67	15.6	15.6	18.7	-
		MBC	37.5	9.35	18.7	9.35	31.2	15.6	37.5	-
*Salmonella typhimurium*		MIC	2.33	4.67	4.67	0.29	1.95	7.8	75	-
		MBC	9.35	4.67	1000	75	62.5	31.2	150	-
*Salmonella enteritidis*		MIC	2.33	9.35	4.67	1.16	-	-	9.35	-
		MBC	600	9.35	2000	300	-	-	9.35	-
*Klebsiella pneumoniae*		MIC	0.58	1.16	0.29	0.29	1.95	7.8	>300	-
		MBC	600	300	400	300	62.5	15.6	>300	-
*Pseudomonas aeruginosa*		MIC	2000	1000	2000	>4000	1000	250	>300	-
		MBC	>4000	1000	4000	>4000	>4000	250	>300	-
*Proteus vulgaris*		MIC	0.29	1000	1.16	1.16	0.49	7.8	150	-
		MBC	2000	>4000	4000	150	7.8	15.6	300	-
*Proteus mirabilis*		MIC	2.33	1000	9.35	1.16	-	-	150	-
		MBC	1000	>4000	>4000	>4000	-	-	300	-
*Aspergillus niger*		MIC	-	150	9.3	18.7	-	-	-	240
		MBC	-	150	9.3	18.7	-	-	-	240
*Aspergillus fumigatus*		MIC	-	300	300	300	-	-	-	240
		MBC	-	300	300	300	-	-	-	240
*Candida albicans*		MIC	-	37.5	37.5	37.5	-	-	-	80
		MBC	-	37.5	37.5	37.5	-	-	-	80
*Penicillium*		MIC	-	18.7	37.5	37.5	-	-	-	80
		MBC	-	18.7	37.5	37.5	-	-	-	80
LD_50_, mg/kg			-	-	-	1420	-	4250	166.7	-

^a^ MIC – minimum inhibitory concentration. ^b^ MBC – minimum bactericide. ^c^ The molecular formula of complexes are reported in Table 1.

## 3. Experimental

### 3.1. Chemistry

All commercially available reagents and chemicals were of analytical- or reagent-grade purity and used as received. ^1^H-NMR and ^13^C-NMR spectra were recorded at room temperature on a Bruker DRX 400 spectrometer in DMSO-d_6_, using TMS as the internal standard. IR spectra were recorded on a Specord-M80 spectrophotometer in the 4000–400 cm^−1^ region using KBr pellets. The chemical elemental analysis for the determination of C, H, N and Br was done the Carlo-Erba LA-118 microdosimeter. Metal ions were determined following the method described by G. Schwarenbach and H. Flaschka [45]. The complexes were studied by thermogravimetry (TG), in a current of air, with a sample heating rate of 1 °C/min, using a SETARAM 92-1600 thermo-balance. Magnetic measurements were carried out on solid complexes using the Gouy’s method [39]. 

X-ray diffraction analysis of compound **5** was carried out on a Nonius KappaCCD diffractometer (MoK*_α_* radiation, λ = 0.71069 Å) at room temperature. The structures of complex **5** was solved by the direct method using SHELXS-86 [46] and SIR-97 [47] software and refined by least squares in the anisotropic approximation for nonhydrogen atoms (CRYSTALS) [48]. The hydrogen atoms were refined isotropically. In complex **5**, all hydrogen atoms were included in the refinement in geometrically calculated positions (except for water molecules in which hydrogen atoms were not located). The C–H and N–H bond lengths varied in the 0.93–0.98 and 0.86–0.89 Å ranges, respectively. The thermal factors U_H_ were taken to be 1.2–1.5 times as high as the U_eq_ values of the carbon and nitrogen atoms. 

#### 3.1.1. General Procedures for the Synthesis of the Schiff Bases **H_2_L^1^**–**H_2_L^10^**

A hot solution of salicylaldehyde (10 mmol) in ethanol (20 mL, 50 °C ) was added to a magnetically stirred solution of H_2_N-NH-C(S)-NH(Y) (10 mmol), where Y = H, in warm ethanol (20 mL). The mixture was refluxed for 1–2 h. The resulting precipitate was filtered, washed with cold ethanol, then with diethyl ether, and dried under vacuum. Crystallization from ethanol gave H_2_L^1^. The same method was applied for the synthesis of H_2_L^2^–H_2_L^10^ by using 2-hydroxybenzaldehyde and its derivatives (X) with thiosemicarbazide or 4-phenylthiosemicarbazide.

*Salicylidene thiosemicarbazone* **(H_2_L^1^**). Yield: 75%. Anal. Calc. (%) for C_8_H_9_N_3_OS (195 g/mol): C, 49.23; H, 4.61; N, 21.53; S, 16.41. Found: C, 49.40; H, 4.52; N, 21.35; S,16.28. IR (cm^−1^, KBr): 3600 (m, OH), 3058 (m, NH), 1560 (s, C=S), 1586 (w, C=N), 1535 (m, NNH), 822 (m, C=S). ^1^H-NMR (DMSO-d_6_, δ, ppm): 11.39 (s, 1H, NNH); 9.88 (s, 1H, OH); 8.37 (s, 1H, HC=N); 7.93, 7.91 (2s, 1H+1H, NH_2_); 8.20, 7.21, 6.85, 6.80 (m, 4H, benzene). ^13^C-NMR (DMSO-d_6_, δ, ppm): 177.6 (C=S); 156.4 (HC=N); 139.6 (C-OH); 116.0, 131.1 120.4, 126.7, 118.9 (benzene).

5-*Chlorosalicylidene thiosemicarbazone* **(H_2_L^2^**). Yield: 70%. Anal. Calc. (%) for C_8_H_8_ClN_3_OS (229.5 g/mol): C, 41.83; H, 3.48; N, 18.30; S, 13.94. Found: C, 42.26; H, 3.34; N, 18.15; S, 13.79. IR (cm^−1^, KBr): 3600 (m, OH), 3058 (m, NH), 1565 (s, C=S), 1585 (w, C=N), 1535 (m, NNH), 820 (m, C=S). ^1^H-NMR (DMSO-d_6_, δ, ppm): 11.44 (s, 1H, NNH); 10.21 (s, 1H, OH); 8.30 (s, 1H, HC=N); 8.16, 8.11 (2s, 1H+1H, NH_2_); 8.10, 7.21, 6.86 , (m, 3H, benzene). ^13^C-NMR (DMSO-d_6_, δ, ppm): 177.8 (C=S); 155.1 (HC=N); 137.7 (C-OH); 117.7, 132.6, 130.4, 122.4, 123.5, 126.5 (benzene). 

5-*Bromosalicylidene thiosemicarbazone* **(H_2_L^3^**). Yield: 71%. Anal. Calc. (%) for C_8_H_8_ BrN_3_OS (274 g/mol): C, 35.03; H, 2.91; N, 15.32; Br, 29.19; S, 11.67. Found: C, 34.89; H, 2.78; N, 15.25; Br, 28.91; S, 11.45. IR (cm^−1^, KBr): 3600 (m, OH), 3055 (m, NH), 1562 (s, C=S), 1584 (w, C=N), 1535 (m, NNH), 823 (m, C=S). ^1^H-NMR (DMSO-d_6_, δ, ppm): 11.42 (s, 1H, NNH); 10.23 (s, 1H, OH); 8.29 (s, 1H, HC=N); 8.21, 8.17 (2s, 1H+1H, NH_2_); 8.21, 7.32, 6.81 (m, 3H, benzene). ^13^C-NMR (DMSO-d_6_, δ, ppm): 177.8 (C=S); 155.6 (HC=N); 137.2 (C-OH); 118.2, 133.2, 111.2, 128.3, 122.9 (benzene). 

5-*Nitrosalicylidene thiosemicarbazone* **(H_2_L^4^**). Yield: 62%. Anal. Calc. (%) for C_8_H_8_N_4_O_3_S (240 g/mol): C, 40.00; H, 3.33; N, 23.33; S, 13.33. Found: C, 40.46; H, 3.12; N, 23.16; S, 13.10. IR (cm^−1^, KBr): 3600 (m, OH), 3058 (m, NH), 1559 (s, C=S), 1586 (w, C=N), 1535 (m, NNH), 821 (m, C=S). ^1^H-NMR (DMSO-d_6_, δ, ppm): 11.53 (s, 1H, NNH); 11.55 (s, 1H, OH); 8.37 (s, 1H, HC=N; 8.29, 8.24 (2s 1H+1H, NH_2_); 8.86, 78.11, 7.04 (m, 3H, benzene). ^13^C-NMR (DMSO-d_6_, δ, ppm): 178.0 (C=S); 161.9 (HC=N); 136.8 (C-OH); 116.5, 126.3, 140.3, 122.2, 121.4 (benzene). 

*5-Methylsalicylidene thiosemicarbazone* **(H_2_L^5^**). Yield: 68%. Anal. Calc. (%) for C_9_H_11_N_3_OS (209 g/mol): C, 51.67; H, 5.26; N, 20.09; S, 15.31. Found: C, 52.02; H, 5.00; N, 19.83; S, 15.04. IR (cm^−1^, KBr): 3600 (m, OH), 3058 (m, NH), 1558 (s, C=S), 1583 (w, C=N), 1535 (m, NNH), 824 (m, C=S). ^1^H-NMR (DMSO-d_6,,_ δ, ppm): 11.50 (s, 1H, NNH); 9.85 (s, 1H, OH); 8.31 (s, 1H, HC=N); 8.02, 8.07 (2s 1H+1H, NH_2_); 7.22, 6.85, 6.62 (m, 3H, benzene); 2.30 (s, 3H, CH_3_). ^13^C-NMR (DMSO-d_6_, δ, ppm): 178.2 (C=S); 153.4 (HC=N); 140.3 (C-OH); 116.0, 133.4, 130.8, 130.5, 118.0 (benzene); 20.9 (CH_3_). 

*3,5-Dichlorosalicylidene thiosemicarbazone* **(H_2_L^6^**). Yield: 75%. Anal. Calc. (%) for C_8_H_7_ Cl_2_N_3_OS (264 g/mol): C, 36.36; H, 2.65; N, 15.90; Cl, 26.89; S, 12.12. Found: C, 36.53; H, 2.48; N, 15.73; Cl, 26.57; S, 11.98. IR (cm^−1^, KBr): 3600 (m, OH), 3058 (m, NH), 1558 (s, C=S), 1587 (w, C=N), 1535 (m, NNH), 822 (m, C=S). ^1^H-NMR (DMSO-d_6_, δ, ppm): 11.48 (s, 1H, NNH); 10.3 (s, 1H, OH); 8.35 (s, 1H, HC=N); 7.98, 7.93 (2s 1H+1H, NH_2_); 7.28, 7.13, (m, 2H, benzene). ^13^C-NMR (DMSO-d_6_, δ, ppm): 177.9 (C=S); 154.0 (HC=N); 140.5 (C-OH); 123.0, 133.1, 126.9, 127.1, 121.5 (benzene). 

*3,5-Dibromosalicylidene thiosemicarbazone* (**H_2_L^7^**). Yield: 72%. Anal. Calc. (%) for C_8_H_7_Br_2_N_3_OS (353 g/mol): C, 27.19; H, 1.98; N, 11.89; Br, 45.32; S, 9.06. Found: C, 27.40; H, 1.78; N, 11.68; Br, 45.03; S, 8.83. IR (cm^−1^, KBr): 3650 (m, OH), 3058 (m, NH), 1560 (s, C=S), 1586 (w, C=N), 1535 (m, NNH), 819 (m, C=S). ^1^H-NMR (DMSO-d_6_, δ, ppm): 11.45 (s, 1H, NNH); 10.55 (s, 1H, OH); 8.29 (s, 1H, HC=N); 8.10, 8.01 (2s, 2H, NH_2_); 8.20, 7.56 (d, 2H, benzene). ^13^C-NMR (DMSO-d_6_, δ, ppm): 178.5 (C=S); 155.4 (HC=N); 150.2 (C-OH); 118.1, 137.5, 111.2, 130.8, 123.0 (benzene). 

*Salicylidene-4-phenylnthiosemicarbazone* **(H_2_L^8^**). Yield: 58%. Anal. Calc. (%) for C_14_H_13_N_3_OS (271 g/mol): C, 61.99; H, 4.79; N, 15.49, S, 11.80. Found: C, 62.27; H, 4.58; N, 15.28; S, 11.73. IR (cm^−1^, KBr): 3600 (m, OH), 3060 (m, NH), 1565 (s, C=S), 1586 (w, C=N), 1535 (m, NNH), 823 (m, C=S). ^1^H-NMR (DMSO-d_6_, δ, ppm): 11.78 (s, 1H, NNH); 9.98 (s, 1H, OH); 8.50 (s, 1H, HC=N); 10.06 (1s 1H, NH-C_6_H_5_); 8.10, 7.22, 6.90, 6.88 (m, 4H, benzene-OH); 7.38, 7.34, 7.34, 7.25, 7.25 (m, 5H-benzene-NH). ^13^C-NMR (DMSO-d_6_, δ, ppm): 177.2 (C=S); 157.1 (HC=N); 140.5 (C-OH); 116.5, 131.8, 120.7, 128.5, 118.4 (benzene-OH); 139.6 (C-NH); 127.5, 127.5, 126.1, 126.1, 127.7 (benzene-NH). 

*5-Bromosalicylidene-4-phenylthiosemicarbazone* **(H_2_L^9^**). Yield: 70%. Anal. Calc. (%) for C_14_H_12_BrN_3_OS (350 g/mol): C, 48.00; H, 3.42; N, 12.00; Br, 22.85; S, 9.14. Found: C, 48.39; H, 3.25; N, 11.80; Br, 22.63; S, 9.00. IR (cm^−1^, KBr): 3600 (m, OH), 3060 (m, NH), 1565 (s, C=S), 1586 (w, C=N), 1535 (m, NNH), 822 (m, C=S). ^1^H-NMR (DMSO-d_6_, δ, ppm): 11.82 (s, 1H, NNH); 10.32 (s, 1H, OH); 8.42 (s, 1H, HC=N); 10.20 (1s 1H, NH-C_6_H_5_); 8.35, 7.35, 6.85 (m, 3H, benzene-OH); 7.38, 7.39, 7.52, 7.51, 7.24 (m, 5H-benzene-NH). ^13^C-NMR (DMSO-d_6_, δ, ppm): 176.6 (C=S); 156.2 (HC=N); 139.7 (C-OH); 118.6, 138.4, 111.6, 133.9, 123.2 (benzene-OH); 139.4 (C-NH); 128.5, 128.5, 126.9, 126.9, 125.9 (benzene-NH). 

*5-Nitrosalicylidene-4-phenylthiosemicarbazone* **(H_2_L^10^**). Yield: 76%. Anal. Calc. (%) for C_14_H_12_N_4_O_3_S (316 g/mol): C, 53.16; H, 3.79; N, 17.72; S, 10.12. Found: C, 53.34; H, 3.57; N, 17.58; S, 9.97. IR (cm^−1^, KBr): 3600 (m, OH), 3060 (m, NH), 1565 (s, C=S), 1586 (w, C=N), 1535 (m, NNH), 822 (m, C=S). ^1^H-NMR (DMSO-d_6_, δ, ppm): 11.91 (s, 1H, NNH); 11.67 (s, 1H, OH); 8.49 (s, 1H, HC=N); 10.35 (1s 1H, NH-C_6_H_5_); 8.98, 8.15, 7.06 (m, 3H, benzene-OH); 7.37, 7.39, 7.31, 7.52, 7.21 (m, 5H-benzene-NH). ^13^C-NMR (DMSO-d_6_, δ, ppm): 176.6 (C=S); 156.2 (HC=N); 139.7 (C-OH); 118.6, 138.4, 111.6, 133.9, 123.2 (benzene-OH); 139.4 (C-NH); 128.5, 128.5, 126.9, 126.9, 125.9 (benzene-NH).

#### 3.1.2. General Procedure for the Preparation of Complexes **1**–**32**

*Synthesis of compound* **1**. 30 mL of ethanolic solution, which contains 10 mmol of salicyliden thiosemicarbazone is mixed with 10 mmol of CuSO_4_·5H_2_O, dissolved in 20 mL of distilled water. The reaction mixture is heated (50–55 °C) and stirred continuously for 1.5 h. The green colored solid, which separated on cooling, was filtered, washed with ethanol, diethyl ether and dried in air. Method for the synthesis of compound **1** is similar to that of work [26] but were modified working conditions.

*Synthesis of Compounds* **2–14**. This compounds have been synthesized according to the above described procedure, using CuSO_4_·5H_2_O or Cu(NO_3_)_2_·3H_2_O and salicyliden thiosemicarbazone, 5-chloro-, 5-bromo-, 5-nitro- salicyliden thiosemicarbazones or 5-bromo-, 5-nitro-salicyliden-4-phenylthiosemicarbazones, in 1:1 molar ratio.

*Synthesis of Compound* **15**. To CuCl_2_·2H_2_O (10 mmol) dissolved in 20 mL ethanol was added salicyliden thiosemicarbazone (10 mmol) dissolved in 15 mL hot ethanol.The mixture was stirred continuously (1 h) and then pyridine alcoholic solution is added till pH=7.5–8. The dark green microcrystals was filtered, washed with ethanol, diethyl ether and dried in air.

*Synthesis of Compound* **16–32***.* This compounds have been synthesized according to the above described procedure, using as initial substances CuCl_2_·2H_2_O, thiosemicarbazones H_2_L^2−7^ and ethanolic solution of pyridine, 2-, 3-, 4-picoline, streptocide (Str), sulphacil (Sfc), norsulphazol (Nor), ethazol (Etz) or sulphadimezine (Sdm), in 1:1:1 molar ratio. The elemental analysis confirms the molecular formula. The physical and analytical data are presented in Table 1.

### 3.2. Cytotoxicity Assay

#### 3.2.1. Preparation of Test Solutions

Stock solutions of the investigated compounds (**H_2_L^1^**–**H_2_L^10^**) and copper complexes **1**–**30** were prepared in dimethylsulfoxide (DMSO) at a concentration of 10 mM and diluted with nutrient medium to various working concentrations. DMSO was used instead of ethanol due to solubility problems.

#### 3.2.2. Cell Culture

Human promyelocytic leukemia cells HL-60 (ATCC, Rockville, MD, USA) were routinely grown in suspension in 90% RPMI-1640 (Sigma, Saint Louis, USA) containing *L*-glutamine (2 nM), antibiotics (100 IU penicillin/mL, 100 µg streptomycin/mL) and supplemented with 10% (v/v) foetal bovine serum (FBS), in a 5% CO_2_ humidified atmosphere at 37 °C. Cells were currently maintained in continuous exponential growth with twice a week dilution of the cells in culture medium [9]. 

#### 3.2.3. Cell Proliferation Assay

The cell proliferation assay was performed using 3-(4,5-dimethylthiazol-2-yl)-5-(3-carboxymethoxyphenyl)2-(4-sulfophenyl)-2H-tetrazolium (MTS) (Cell Titer 96 Aqueous, Promega, Madison, Wi, USA), which allowed us to measure the number of viable cells. In brief, triplicate cultures of 1 x 10^4^ cells in a total of 100 µL medium in 96-well microtiter plates (Becton Dickinson and Company, Lincoln Park, NJ, USA) were incubated at 37 °C, 5% CO_2_. Compounds were dissolved in DMSO to prepare the stock solution of 1 × 10^−2^ M. These compounds were diluted at the appropriate concentration (1 or 10 µM) with culture media, added to each well and incubated for 3 days. Following each treatment, 20 µL MTS was added to each well and incubated for 4 h. MTS is converted to water-soluble coloured formazan by dehydrogenase enzymes present in metabolically active cells. Subsequently, the plates were read at 490 nm using a microplate reader (Molecular Devices, Sunnyvale, CA). The results were reported as the percentage of cell proliferation inhibition compared to the control (basal cell proliferation=100%).

### 3.3. Antibacterial Activity

The antibacterial activity of complexes and also of their prototype Furaciline has been determined under liquid nutritive environment [2% of peptonate bullion (pH 7.0)] using successive dilutions method [36,37,38]. *Staphylococcus aureus (Wood-46, Smith, 209-P), Staphylococcus saprophyticus, Streptococcus faecalis, Escherihia coli (O-111), Salmonella typhimurium, Salmonella enteritidis, Klebsiella pneumoniaie, Pseudomonas aeruginosa, Proteus vulgaris* and *Proteus mirabilis* standard stems were used as reference culture for *in vitro* experiments. The dissolution of studied substances in dimethylformamide, microorganisms cultivation, suspension obtaining, determination of minimal inhibition concentration (MIC) and minimal bactericide concentration (MBC) have been carried out according to the method previously reported [39].

### 3.4. Antifungal Bioassay

Antimycotic properties of the complexes were investigated *in vitro* on laboratory stems: *Aspergillus niger, Aspergillus fumigatusi, Candida albicans* and *Penicillium.* The activity has been determined in liquid Sabouroud nutritive environment (pH 6.8). The inoculates were prepared from fungi stems which were harvested during 3–7 days. Their concentration in suspension is (2–4) × 10^6^ colonies form unities in milliliter. Sowings for levures and micelles were incubated at 37 °C during 7 and 14 days, respectively.

## 4. Conclusions

Ten new salicyliden thiosemicarbazones ligands and their corresponding Cu(II), Ni(II) and Zn(II) complexes have been synthesized and characterized. The molecular structure of the complex **5** has been determined by single crystal X-ray diffraction method. The IR, ^1^H-NMR and ^13^C-NMR data were successfully used to elucidate the formation of the salicyliden thiosemicarbazones ligands. All ligands and their metal complexes were tested as inhibitors of HL-60 cells proliferation. The ligands have unsignificant inhibitor activity at 0.1 and 1.0 μmol/L, but at 10 μmol/L **H_2_L^8^**, **H_2_L^9^** and **H_2_L^10^** inhibit the cell proliferation. The copper complexes, including inner sphere water and tridentate ONS ligands, are more active than those containing inner sphere amine, which blocked the metal active centre. The most indicative criteria for future synthesis of biological active coordination compounds from theview point of the inhibition of HL-60 cell proliferation, antibacterial and antifungal activity: use of copper (II) complexes and presence of sulphur atom in the tridentate organic ligand.

## Supplementary Data

CCDC 623449 contain the supplementary crystallographic data for C_16_H_8_Br_2_Cu_2_N_6_O_12_S_3_ (**5**). These data can be obtained free of charge via http://www.ccdc.cam.ac.uk/conts/retrieving.html, or from the Cambridge Crystallographic Data Centre, 12 Union Road, Cambridge CB2 1EZ, UK; fax: (+44) 1223-336-033; or e-mail: deposit@ccdc.cam.ac.uk.
